# Risk factors for colonization and infection by multiresistant bacteria

**DOI:** 10.1186/2197-425X-3-S1-A126

**Published:** 2015-10-01

**Authors:** S Carvalho Brugger, R Gavilan Rabell, M Miralbes Torner, G Jimenez Jimenez, S Iglesias Moles, J Trujillano Cabello, M Vallverdú Vidal, B Balsera Garrido, F Barcenilla Gaite, M Palomar Martínez

**Affiliations:** Hospital Universitari Arnau de Vilanova, Lleida, Spain; Hospital Universitari Arnau de Vilanova., Lleida, Spain

## Introduction

In 2013, the “Zero Resistance” (RZ) program was launched in Spain, to help prevent the emergence of multiresistant bacteria (MRB) in critically ill patients. One of its recommendations is to complete a checklist upon patient admission in Intensive Care Unit (ICU) to identify those patients at high risk for colonization or infection by MRB*.

## Objectives

To analyse the effectiveness of the checklist proposed as a way to early detect MRB and the role of other comorbidities.

## Methods

A prospective study from March 17^th^, 2014 to January 31^rst^, 2015. All patients admitted to a polyvalent ICU of a general hospital were submitted to the checklist proposed, with the application of contact precaution (CP) strategies for patients with risk factors (RF) for colonization or infection by MRB. Bacteriologic swabs (nasal, pharyngeal, axillary and rectal) were routinely performed on all patients admitted, besides diagnostic cultures when they were necessary. Furthermore, we analysed other pathological variables and comorbidities (diabetes, renal failure, immunosuppression state, neoplasia, cirrhosis, chronic obstructive pulmonary disease -COPD-, organ transplantation, malnutrition and type of admission to ICU - urgent or programmed). Univariate and multivariate analysis of risk factors for MRB with binary logistic regression were performed. Statistical significance was set at CI 95%.

## Results

780 patients were admitted. 231 (29,6%) met some CP criteria. In 78 (10%) were detected one or more MRB, 49 of these (62,8%) presented CP criteria according to the checklist. 23 met 1 criteria, 17 met 2 criteria and 10 met 3 or more criteria with accumulation of risk. In 29 (37, 2%) MRB carriers it was not identified any of the RF from the checklist. Tables 1 and 2 show RF and comorbidities that were significant as added risk for MRB. We did not find significant relation between renal failure, immunosuppression state, neoplasia, cirrhosis or organ transplantation and being MRB carrier.

**Table 1:** RZ checklist.

RZ CHECK LIST	OR (IC 95%) UNIVARIATE	OR (IC 95%) MULTIVARIATE
Hospitalization >5 days in prior 3 months	3,2 (2,0-5,2)	2,05 (1,1-3,7)
Institutionalized patient	3,7 (1,6-8,6)	NS
Prior MRB colonization/infection	14 (5,7-34,4)	8,7 (3,3-22,9)
Antibiotherapy >7 days in prior month	4,2 (2,5-7,2)	2,1 (1,1-4,1)
Chronic kidney disease with dialysis	1,5 (0,2-12,7)	NS
Colonization susceptibility (bronchiectasis, cystic fibrosis)	2,0 (0,7-5,5)	NS

**Table 2:** Comorbidities and type of admission.

COMORBIDITY	OR (IC 95%) UNIVARIATE	OR (IC 95%) MULTIVARIATE
DM type II	1,9 (1,2-3,1)	1,8 (1,1-3,1)
COPD	1,9 (1,1-3,4)	NS
Malnutrition	1,9 (1,1-3,6)	NS
Urgent admission to ICU	1,85 (1,1-3,0)	NS

## Conclusions

In our environment, the checklist did not detect 37% of patients with MRB. Prior antibiotic therapy and prior colonization were the best predictors. Further factors as diabetes could help on detection of MRB in ICU.
